# Supplementary material for: A Correlational Analysis of Phthalate Exposure and Thyroid Hormone Levels in Common Bottlenose Dolphins (Tursiops truncatus) from Sarasota Bay, Florida (2010–2019)
Source: Animals (Basel). 2022 Mar 24;12(7):824. doi: 10.3390/ani12070824 (PMC8996861; doi:10.3390/ani12070824)
Supplement: Supplementary file 1 [file animals-12-00824-s001.zip › animals-1613149-supplementary.pdf]

Supplemental Materials

Table S1. Kendall's tau correlation coefficient and *p*-values between mono(2-ethylhexyl) phthalate and hormones in common bottlenose dolphins (*Tursiops truncatus*) sampled from Sarasota, Florida (2010-2019). Statistical significance observed at α= 0.05, as indicated by the values in **bold** (*n*= 50). Kendall's tau (*p*)

Group	Triiodothryonine (T3)	Total thyroxine (T4)	Free thyroxine (FT4)
All mono(2-ethylhexyl) phthalate (MEHP; *n*= 50)	0.06 (0.52)	-	-
All Adults (*n*= 33)	-	-0.002 (1.00)	-
Adult Female MEHP (*n*= 17)	-	-	**0.36 (0.04)**
Adult Male MEHP (*n*= 16)	-	-	**0.42 (0.02)**
All Juveniles (*n*= 17)	-	0.03 (0.93)	-
Juvenile Female MEHP (*n*= 12)	-	-	-0.15 (0.58)
Juvenile Male MEHP (*n*= 5)	-	-	0.20 (0.79)

Table S2. Power analysis for correlations between mono(2-ethylhexyl) phthalate (MEHP) and hormones in urine sampled from common bottlenose dolphins (*Tursiops truncatus*) sampled from Sarasota, Florida during 2010-2019.

Group	Small Effect Size	Medium Effect Size	Large Effect Size
Adults (*n*= 33)	0.087	0.41	0.87
Juveniles (*n*= 17)	0.065	0.21	0.53
Females (*n*= 29)	0.081	0.36	0.81
Males (*n*=21)	0.071	0.27	0.66
Adult Females (*n*= 17)	0.067	0.23	0.57
Adult Males (*n*= 16)	0.065	0.21	0.53
Juvenile Females (*n*= 12)	0.059	0.15	0.37
Juvenile Males (*n*= 5)	0.056	0.081	0.14

Table S3. Analyte detection frequency by demographic group for common bottlenose dolphins (*Tursiops truncatus*) sampled from Sarasota, Florida during 2010-2019 given as %.

Analyte	Adult Female % (*n*= 17)	Adult Male % (*n*= 16)	Juvenile Female % (*n*= 12)	Juvenile Male % (*n*= 5)
Mono(2-ethylhexyl) phthalate (MEHP)	61.11	43.75	63.64	60.00
Triiodothyronine (T3)	66.67	43.75	63.64	60.00
Total thyroxine (T4)	100.00	100.00	100.00	100.00
Free thyroxine (FT4)	100.00	100.00	100.00	100.00
